# Chronic Inflammatory Stenosis of the Cardiac Region of the Oesophagus

**Published:** 1913-11

**Authors:** 


Chronic Inflammatory Stenosis of the Cardiac Region of the Oesophagus. G. Liebault, *Rev. Hebd. de Laryn.*, May 10, 1913. These occur usually in adults from trivial causes, affecting the œsophagus at its normal retraction as it passes through the diaphragm. The interaction of an erosion and the resulting spasm are compared to the phenomena of anal fissure. The diagnosis, especially to exclude neoplasms, etc., need not be discussed here. The prognosis is good—which scarcely agrees with the recommendation of a preliminary gastrotomy.

